# Analecta Minora

**Published:** 1825-10-01

**Authors:** 


### ANALECTA MINORA,

OR

### *MINOR PERISCOPE.*

sparsa Colligens

1. *Application of Leeches.** There is sometimes considerable difficulty in persuading these little vampyres to do their duty—and particularly to do it in the very place where we wish it to be done. It appears that the more humble limbs of the Profession in Naples, namely the Barber-surgeons, have

* Dr. Milne, resident Physician at Naples.

a talisman which is sure to make the leech implicitly obey the mandate of his master. This talisman is neither more nor less than a feather plucked from the tail or wing of a young pigeon (a bird which they rear for this purpose) with the spongy bloody point of which they touch the part where they wish the bite to be made. They then apply the leech which instantly and invariably makes the aperture on that spot. By this manœuvre they plant, in a few minutes, any number of leeches on any given point of the body's surface.

---

2. *Ovarian Dropsy.* The attention of the Profession is now considerably excited towards the treatment of this opprobrium medici et chirurgi, in consequence of the cases recently published by Mr. Lizars. The following retrospection may, therefore, not be unacceptable at the present moment.

In the year 1724, Mr. Houston published, in the 33d volume of the Philosophical Transactions, a case of ovarian dropsy cured by a surgical operation. Mr. H. first introduced a trocar, but found that the contents would not flow. He then made an incision with a scalpel, five inches in length, but still the fluid was of too consistent a nature to make its issue even through such an opening. Mr. Houston, therefore, had recourse to an instrument something like a large spoon, by means of which he dug out the contents of the cyst, being partly of a pultaceous, partly of a gellatinous consistence. An immense mass was thus removed, after which he brought the sides of the wound together, and secured them by suture, leaving an aperture, however, through which there continued, for some time, a discharge from the cyst. Finally, the wound closed entirely, and the woman was perfectly cured.

It appears that Le Dran was in the habit, in the latter years of his life, of making large incisions into ovarian dropsical cysts, and keeping them open by means of tents, and even of leaden tubes. He sometimes threw in stimulating injections, and by these means often reduced the cysts to a very small volume, though there always remained a fistulous opening afterwards, except in one case, where the fistula ultimately healed, and the cyst did not again fill.—*Mem. de l'Acad. de Chir. vol.* ii.

Thus we see that instances may be collected from the records of Surgery, where ovarian dropsies have been cured by operations less terrible and dangerous than the total removal of the cyst by gastrotomy. Might it not be advisable to have recourse to these older methods prior, at least, to the new operation?

---

3. *Corpora Lutea.* These were long considered as evidence of impregnation. Latterly they have not been looked upon as unequivocal criteria, and it has been concluded that they may sometimes owe their origin to sensual intercourse or excitement. A fact related by Dr. Beck, in his Medical Jurisprudence, proves that none of the alleged causes are essentially necessary for the production of the phenomena in question. He states that Dr. Mackintosh of Edinburgh and Dr. J. Scott examined the body of a child only *five years* of age, in whom the hymen was perfect, but whose ovaria presented numerous corpora lutea, as distinct as in the adult impregnated female.

---

4. *Mercurial Ointment.* M. Hernandez has proposed to the Society of Pharmacy of Paris, a new mode of preparing the unguentum hydrargyri. It consists in heating the mortar, in which the ointment is to be prepared, so as to liquify the lard. As the lard cools the mercury becomes incorporated with it in a far more speedy manner than by the method now in use.

---

5. *Antidotes.* Antidotes to hydrocyanic acid and opium are said by Mr. John Murray (Ed. Journ of Science) to be positively found in ammonia and acetic (not acetous) acid. This assertion he supports by experiments on animals. As to the hydrocyanic acid we have nothing to say; but in the antidote to opium we should put little faith. The best antidote is the removal of the substance from the stomach; and as this can now be done mechanically, no medical practitioner should be without an apparatus for the purpose, otherwise he runs the risk of injury to his reputation, when the emergency occurs.

☞ As the instrument is rather expensive, and as it is not likely that more than one person shall poison himself at a time in a town, we suggest (what indeed we know to be now put in practice in several places) that two or more medical men in each town should join, and have an apparatus for common use, deposited in a situation where it could always be found without delay.

---

6. *Euphorbia Lathyrus, or Caper Spurge.* We drew the attention of our brethren last quarter to this new purgative, which seems to combine all the good qualities of the oil of croton, without any of its repulsive properties. M. Caventon, we are informed in a late number of the Revue Medicale, has made experiments on the oil of the seeds of this plant, and finds that six or eight drops purge copiously without griping. The oil is so inodorous and tasteless that it may be given to children on sugar or otherwise, without their suspecting physic—no mean advantage. It now costs about ten pence an ounce in Paris, and this quantity is calculated to be sufficient to purge briskly 96 adults! We advise our pharmaceutical brethren to supply themselves as soon as possible with this useful and cheap expurgator of the chylopoietic organs.

---

7. *Variolous Eruption.* Mons. Velpeau has lately read a memoir, before the Royal Academy of Medicine of Paris, tending to prove that, if, during the first two days of the variolous eruption, we cauterise the pustules by means of a probe dipped in solution of lunar caustic, the progress of the pustules will be arrested. If this be done at a later period, the duration of the pustules will be curtailed—and, at all events, the marks of their existence will be entirely prevented. He appeals to Messrs. Bretonneau, Billard Dumeril, and Serres, for evidence of the truth of these assertions.

☞ If this be confirmed by other practitioners, it will be of considerable importance in a cosmetic as well as pathologic point of view.

---

*Dr. Philip to the Editor.*

It was particularly gratifying to Mr. Brodie and myself, that M Breschet and Dr. Edwards of Paris, in a repetition of experiments made by us about four years ago, confirmed the results we had obtained. In a later paper, the same gentlemen have given an account of some additional experiments which have led them to inferences, some of which are in opposition to ours. I shall here make a few observations on these inferences in the order in which they occur in the paper, which is rendered the more necessary by the respectability of the quarter from which they come.

The first is "that the division of the eighth pair of nerves," that is with loss of substance, "considerably retards, but does not arrest, the digestive process."

This operation certainly can have no effect in preventing the action of the gastric fluid secreted before the operation, and which has accumulated in the stomach. But from the facts, that the longer the animal lives after the operation, the less digested food is found in its stomach, and that, if it survives long, the undigested food alone remains in it, points we have ascertained by many trials; and from experiments made on the newly dead animal which are related in my Treatise on the Vital Functions, it appears that the secretion of gastric fluid is immediately arrested by this operation.

2. "That the defect in the digestive process, in the case before us, depends on the paralysis of the muscular fibres of the stomach."

In our experiments we always found that the stomach discharged its digested contents into the intestine as readily after as before the operation.

3. "That the vomitings which often follow the division of the nerves depend on a paralysis of the muscular fibres of the œsophagus."

We always found that the function of the œsophagus was as well performed after, as before the division of the nerves, the animal continuing to swallow with the same ease as in health.

4. "That the re-establishment of the digestion, after the division of the nerves, by electricity, does not depend on any chemical action of that agent, but on its exciting the movements which are necessary for renewing the surface of the alimentary mass, and bringing, by turns, its different parts into contact with the surface of the stomach."

I have just had occasion to remark that we always found that the stomach discharged its digested contents into the intestine as readily after as before the operation. Indeed, if the stomach and bowels be wholly removed, even in the newly dead animal, and thus all their nervous connexions destroyed, their peristaltic motions continue for some time unimpaired.

5. "That by mechanical irritation of the extremity of the lower portion of the divided nerve the same results, though less marked, are obtained as from electricity."

Mr. Cutler, at my request, was so good as to repeat the experiment from which this inference is made on a rabbit. We were both perfectly acquainted with the appearances of the rabbit's stomach under the various circumstances connected with such experiments, and feel no hesitation in saying that, where the mechanical irritation employed by M. Breschet and Dr. Edwards had existed for eight hours, during which the animal lived after the operation, the appearance of the food, which it had eaten just before the operation, was exactly what it would have been from the removal of part of the nerve, had no cause of irritation existed. The stomach was filled with masticated parsley in the state in which it was swallowed. Mr. Brodie also saw the food, and agreed with us.

6. "That the chief function of the pneumogastric nerves, as far as relates to the organs of digestion only, is to regulate the movements of the stomach which accelerate digestion by facilitating the contact of the gastric fluid with the different parts of the alimentary mass."

The reply to this statement is made in what is said of the second and fourth statements.

I remain, dear Sir,
very faithfully yours,
A. P. W. PHILIP.

